# PeRsOnaliSed care Planning for oldER people with frailty (PROSPER): protocol for a randomised controlled trial

**DOI:** 10.1186/s13063-023-07857-1

**Published:** 2024-01-02

**Authors:** Anne Heaven, Peter Bower, Florence Day, Amanda Farrin, Catherine Fernadez, Marilyn Foster, Robbie Foy, Rebecca Hawkins, Claire Hulme, Sara Humphrey, Rebecca Lawton, Catriona Parker, Ellen Thompson, Robert West, Andrew Clegg

**Affiliations:** 1 Academic Unit for Ageing and Stroke Research, University of Leeds, Bradford Institute for Health Research, Bradford Teaching Hospitals NHS Foundation Trust, Bradford, BD9 6RJ UK; 2 NIHR Older People and Frailty Policy Research Unit, Centre for Primary Care and Health Services Research, Manchester Academic Health Science Centre, University of Manchester, M13 9PL, Manchester, UK; 3 Leeds Institute of Clinical Trials Research (LICTR), Clinical Trials Research Unit, University of Leeds, Leeds, LS2 9JT UK; 4 PPI Representative, c/o Academic Unit for Ageing and Stroke Research, University of Leeds, Bradford Institute for Health Research, Bradford Teaching Hospitals NHS Foundation Trust, Duckworth Lane, Bradford, BD9 6RJ UK; 5 Leeds Institute of Health Sciences, School of Medicine, University of Leeds, Leeds, LS2 9JT UK; 6 Health Economics Group, Institute of Health Research, University of Exeter, Exeter, EX1 2LU UK; 7 OPMH & Dementia at Yorkshire and Humber Clinical Network, Rotherham, UK; 8 School of Psychology, University of Leeds, Leeds, LS2 9JT UK; 9 Cancer Clinical Trial Centre, Weston Park Cancer Centre, Whitham Road, Sheffield, S10 2SJ UK

**Keywords:** Personalised-Care-Planning, Quality of life, Older people, Frailty, eFI, RCT

## Abstract

**Background:**

Frailty is common in older age and is characterised by loss of biological reserves across multiple organ systems. These changes associated with frailty mean older people can be vulnerable to sudden, dramatic changes in health because of relatively small problems. Older people with frailty are at increased risk of adverse outcomes including disability, hospitalisation, and care home admission, with associated reduction in quality of life and increased NHS and social care costs. Personalised Care Planning offers an anticipatory, preventative approach to supporting older adults to live independently for longer, but it has not been robustly evaluated in a population of older adults with frailty.

**Methods:**

Following an initial feasibility study, this multi-centre, individually randomised controlled trial aims to establish whether personalised care planning for older people improves health-related quality of life. It will recruit 1337 participants from general practices across Yorkshire and Humber and Mid-Mersey in the North of England. Eligible patients will be aged 65 and over with an electronic frailty index score of 0.21 or above, living in their own homes, without severe cognitive impairment and not in receipt of end-of-life care. Following confirmation of eligibility, informed consent and baseline data collection, participants will be individually randomised to the PeRsOnaliSed care Planning for oldER people with frailty (PROSPER) intervention or usual care in a 2.6:1 allocation ratio. Participants will not be blinded to allocation, but data collection and analysis will be blinded. The intervention will be delivered over 12 weeks by a Personal Independence Co-ordinator worker based within a voluntary sector organisation, Age UK. The primary outcomes are health-related quality of life, measured using both the physical and mental components of the Short-Form 12 Item Health Questionnaire at 12 months after randomisation. Secondary outcomes comprise activities of daily living, self-management capabilities and loneliness, admission to care homes, hospitalisations, and health and social care resource use at 12 months post randomisation. Parallel cost-effectiveness and process evaluations will be conducted alongside the trial.

**Discussion:**

The PROSPER study will evaluate the effectiveness and cost-effectiveness of a personalised care planning approach for older people with frailty and inform the process of its implementation.

**Trial registration:**

ISRCTN16123291.  Registered on  28 August 2020.

**Supplementary Information:**

The online version contains supplementary material available at 10.1186/s13063-023-07857-1.

## Background and rationale

Frailty is a condition characterised by reduced biological reserves and increased vulnerability to adverse outcomes [1]. Frailty is considered more readily reversible at early stages than disability and has higher predictive value than chronic disease for adverse outcomes. Single disease management frameworks are less relevant for older people living with frailty and best practice suggests that care for older people with frailty should be proactive and person-centred, responsive to personal experiences of illness, individual priorities, and predicaments [1, 2].

Personalised Care Planning (PCP) is an anticipatory, negotiated series of guided conversations between a patient and a suitably trained individual [3] and presents a promising way to achieve a shift towards proactive, person-centred care in frailty [2–4]. PCP moves away from a focus on individual disease management and a reactive crisis-driven response in healthcare. Shared decision making is the central mechanism in PCP involving collaborative discussions between patients and practitioners, goal setting, and agreement of an action plan. This process enables linkage to additional mechanisms for improving outcomes through more effective self-management, better care coordination, and better access to community resources (social support). It includes creating a care plan and monitoring delivery through regular follow-up. Key outcomes are improved physical and mental health, self-management capabilities, health-related behaviours, and changes in health service use [3].

Social cognitive theory (SCT) is the theoretical model that resonates most with the tenets of PCP in the context of frailty and provides the underpinning theory for developing and optimising our planned intervention [5]. SCT specifies factors governing the acquisition of competencies that can profoundly affect physical and emotional wellbeing [6]. It identifies knowledge, skills, self-efficacy, outcome expectations, goals, and concrete plans, as well as the perceived social and environmental facilitators and impediments as core determinants influencing our health habits. Social and environmental factors are of core importance for maintaining quality of life in older age and are therefore highly relevant for optimising PCP for older people with frailty.

Although a 2015 Cochrane review identified that PCP for long-term conditions (LTCs) can improve physical and mental health, and self-management capability, the majority of the 16 studies summarised were focused on single LTCs such as diabetes [3]. None of the studies selected participants based on frailty. Furthermore, a comprehensive evaluation of PCP in the UK has identified widespread, poor implementation [7, 8].

In England, the 2019 National Health Service (NHS) Long Term Plan set out key ambitions for the health service over the next 10 years [9]. The Plan included a focus on enabling older people living with frailty to live independently at home for longer. This was to be achieved via primary care-based multidisciplinary teams providing tailored support. Any intervention therefore must be sufficiently robust and flexible to integrate with commissioning and provider organisations.

In 2013 Age UK—the largest voluntary sector organisation providing support and services to older adults in the UK—developed an Integrated Personalised Care Planning (IPCP) service, working across commissioning and provider organisations [10]. We collaborated with the Age UK IPCP service to develop our PROSPER (Personalised Care Planning for Older People) intervention—designed to improve quality of life for older people with frailty and reduce use of health and social care services.

The trial methods, fidelity, and implementation were tested in a feasibility study in 2019 [11] and subsequent adjustments made to both the trial design and intervention delivery. Here we describe the protocol for the definitive trial evaluation of PROSPER compared with usual care (UC) to evaluate its effectiveness in improving quality of life (QoL) for older people with frailty.

## Aim and objectives

This trial aims to evaluate the clinical and cost-effectiveness of PCP for older people with frailty, compared with UC alone.

The primary objective is to establish whether PCP for older people with frailty improves health-related quality of life (HRQoL), measured using either the Physical Component Summary (PCS) or the Mental Component Summary (MCS) of the Short-Form 12-Item Health Questionnaire (SF12) at 12 months after randomisation.

Secondary objectives are to establish:

1. Whether PCP improves activities of daily living at 12 months post-randomisation.
2. Whether PCP improves self-management capabilities and loneliness 12 months post-randomisation.
3. Whether PCP reduces admission to care homes at 12 months post-randomisation.
4. Whether PCP reduces hospitalisations, overall health, and social care resource use at 12 months post-randomisation.
5. The cost-effectiveness of PCP.
6. The process of implementation within participating sites and determine how PCP should be implemented more broadly across the wider NHS.

## Methods

### Trial design

PROSPER is a pragmatic, multi-centre, randomised controlled trial (RCT) with a two-level partially nested hierarchical design, comparing PCP versus UC in older people with frailty. The previous cluster randomised feasibility trial indicated that PCP was being individually tailored by Personal Independence Coordinator (PIC) workers in partnership with older people, with limited involvement of the primary care clinical team, minimising risks of intervention contamination. This aligns with the overall aim and theoretical underpinnings of PROSPER to direct a shift away from a medical model of care in frailty towards a more socially orientated model reflecting how the intervention would likely be implemented in practice. Considering this information, we have changed the unit of randomisation from a cluster to an individually randomised controlled trial. The change in trial design will also help mitigate some of the challenges faced during recruitment in the feasibility study, allowing further detail about the intervention to be provided up-front, with the potential advantage of minimising the number of participants who subsequently decline participation in the early stages of the intervention. Additional benefits include smaller overall sample size and less constraint in terms of recruiting larger numbers from individual practices.

This study will also comprise a mixed method embedded process evaluation and Study Within a Trial (SWAT). These will be reported separately.

### Study setting

Participants will be recruited from general practices within four geographic localities in the relatively rural Northwest of England (Mid-Mersey region) and a large area of conurbation in West Yorkshire (Leeds, Bradford, Wakefield).

### General practice recruitment and eligibility

A range of approaches will be used to engage with general practices including local National Institute for Health Research (NIHR) Clinical Research Networks (CRNs), professional networks, and practices listed on the Office for Health Improvement and Disparities (OHID) website [12]. General practice eligibility will be based on the following:

- Having completed a Site Feasibility Assessment Questionnaire (SFQ), followed by a meeting.
- Provided confirmation of capacity and capability (C&C).
- Using either SystmOne or Egton Medical Information Systems (EMIS) for patient electronic health records (EHRs).
- Having identified a member of staff with a clinical interest in older people or frailty to act as a ‘champion’.
- Allowing research staff with appropriate permissions to access participant EHRs.
- Being willing to make reminder calls/send SMS reminders to patients who do not respond to an invitation letter.
- Located within the geographical footprint of our voluntary sector (Age UK) delivery teams.
- Willing to engage with intervention delivery staff from Age UK.
- Assigning a practice ‘buddy’ to the Age UK team for administrative purposes.
- Providing an honorary contract for Age UK delivery team to allow access to EHRs of study participants.

Practices that provide an existing or planned PCP service targeting older people with frailty that significantly overlaps with the PROSPER intervention model will be excluded.

### Participant identification and eligibility

Potential participants will be identified by practice staff electronically screening for the inclusion/exclusion criteria within patient EHRs. Templates to assist with searches will be available for the two most used EHR systems, EMIS and SystmOne.

#### Inclusion criteria

We undertook initial statistical and health economic modelling work to inform how PCP should be targeted, based on the relationship between frailty (measured using the electronic frailty index (eFI)), health and social care use, and health-related quality of life, using existing secondary data sources [13, 14]. Patients meeting *all* the following criteria (and none of the exclusion criteria) will be eligible for inclusion:

- Frailty defined by eFI range ≥0.21 (EHR screen)
- Aged 65 or over (EHR screen)
- Willing and able to give informed consent (or has a personal consultee declaration if the patient lacks capacity; assessed at baseline visit)

#### Exclusion criteria

Patients meeting *any* of the following criteria will not be eligible for inclusion, assessed at screening:

- Resident of a care home (EHR screen).
- Registered on Gold Standards Framework, indicating an individual is likely to be in the last year of life (GSF; EHR screen).
- Deemed inappropriate to approach for safety reasons (GP/Advanced Nurse Practitioner (ANP) screen).
- Severe cognitive impairment, as participants must be able to engage with goal setting and action planning, defined as a Blind Montreal Cognitive Assessment (MoCA) score of <7 assessed at telephone registration, or full MoCA score <10, assessed face-to-face at baseline visit due to mental capacity concerns.
- Member of household currently or previously consented to take part in this study (assessed at registration).
- In receipt of another PCP service for older people (assessed at registration).

Eligibility waivers to participant inclusion/exclusion criteria are not permitted. The number of eligible participants and the number of potential participants removed, along with the reason will be logged by the University of Leeds Clinical Trials Research Unit (UoL CTRU) where possible. Practices will provide an anonymised list of the gender, age, eFI, ethnicity, and invitation number to allow comparison of the characteristics of those declining to participate to those entering the trial. This information will support discussion of generalisability of study results in accordance with CONSORT reporting guidelines [15]. Participant eligibility lists will be generated once. However, if the response rate is low, potential participants will be re-approached 3–6 months after their first invitation after the lists have been reviewed and any potential participant who has left the practice or died in the interim removed.

### Recruitment

An information pack will be sent out by the practice staff to all those on the eligibility list in batches, if necessary. The pack will include:

- An invitation letter for the participant
- An information sheet for the participant
- An invitation reply form
- A pre-paid envelope

The information provided will summarise the study and ask if the potential participant would be willing to consider participation in the study. The information will state potential participants will be contacted by the study team if no response is received within 7 days. Practices may also send a single SMS text reminder.

Contact details for the research team will be provided should the potential participant require more information before committing to the study. In addition, this can be indicated on the enclosed reply form. The completed reply form (including an option to decline, with or without noting a reason) can be returned directly to the research team using a pre-paid envelope or alternatively potential participants or consultees can respond via telephone, email, or request a return call via SMS.

It may not be clear at the time of approach whether the potential participant lacks capacity to consent for themselves so information that can be given to a potential personal consultee will be available at the consent/baseline visit.

The above process will be repeated until the recruitment target is met. Reasons for decline will be documented when available. Study materials will be re-sent if requested, but no further calls will be made unless specifically requested by the patient.

### Trial consent, assent, and randomisation

A researcher trained in Good Clinical Practice (GCP) and with an appropriate level of Disclosure Barring Service (DBS) check, will initially telephone potential participants who have returned an expression of interest (EOI) from the study invitation. *Verbal* consent will be obtained over the telephone. Final eligibility checks will be done, and the potential participant’s details will be collected (practice name, date of birth) so they can be registered onto the study. Following registration, the researcher will arrange a visit to the potential participant’s home to discuss the study further, obtain *written* informed consent, confirm eligibility, and collect baseline data. Brief information about the process evaluation and SWAT will be given at this time but these will have separate recruitment, randomisation, and consent procedures.

A lack of capacity to consent is not a basis for exclusion. If a researcher has concerns about capacity of the potential participant to provide verbal consent, they will arrange a home visit to undertake a capacity assessment in line with the Mental Capacity Act (2005) guidance [16]. At this time, a consultee will be identified, and advice sought, if necessary. The MoCA test will be administered separately. Participants who record a MoCA score of <10 face-to-face will be excluded from the study, as they are unlikely to be able to engage in meaningful shared decision making with the PIC. Baseline data will not be collected for those who are ineligible. Research staff will also ascertain whether a face-to-face visit or telephone support will be needed for follow-up at the baseline visit.

#### Consent

The right to decline consent to participate in the study, or withdraw from the study, without providing a reason, at any time will be respected. Reasons for declining participation and withdrawals will be noted, if provided.

#### Potentially vulnerable adults

Researchers will review capacity as previously described and involve friends and family as ‘chaperones’ when requested. Where individuals have specific impairments such as sight or hearing loss, the research staff will take measures to mitigate these, such as assisted completion, visual prompts, and use of the Blind MoCA. Research staff will consider participant fatigue during data collection and offer additional visits, if required.

#### Safeguarding of adults

All research staff will receive training in recognising and responding to abuse and will follow their organisational safeguarding procedures to escalate incidents via their individual line managers. In addition, GPs will be notified by the CTRU if a participant’s Geriatric Depression Score (GDS) score at baseline or follow-up indicates possible depression.

#### Randomisation

**Fig. 1:**
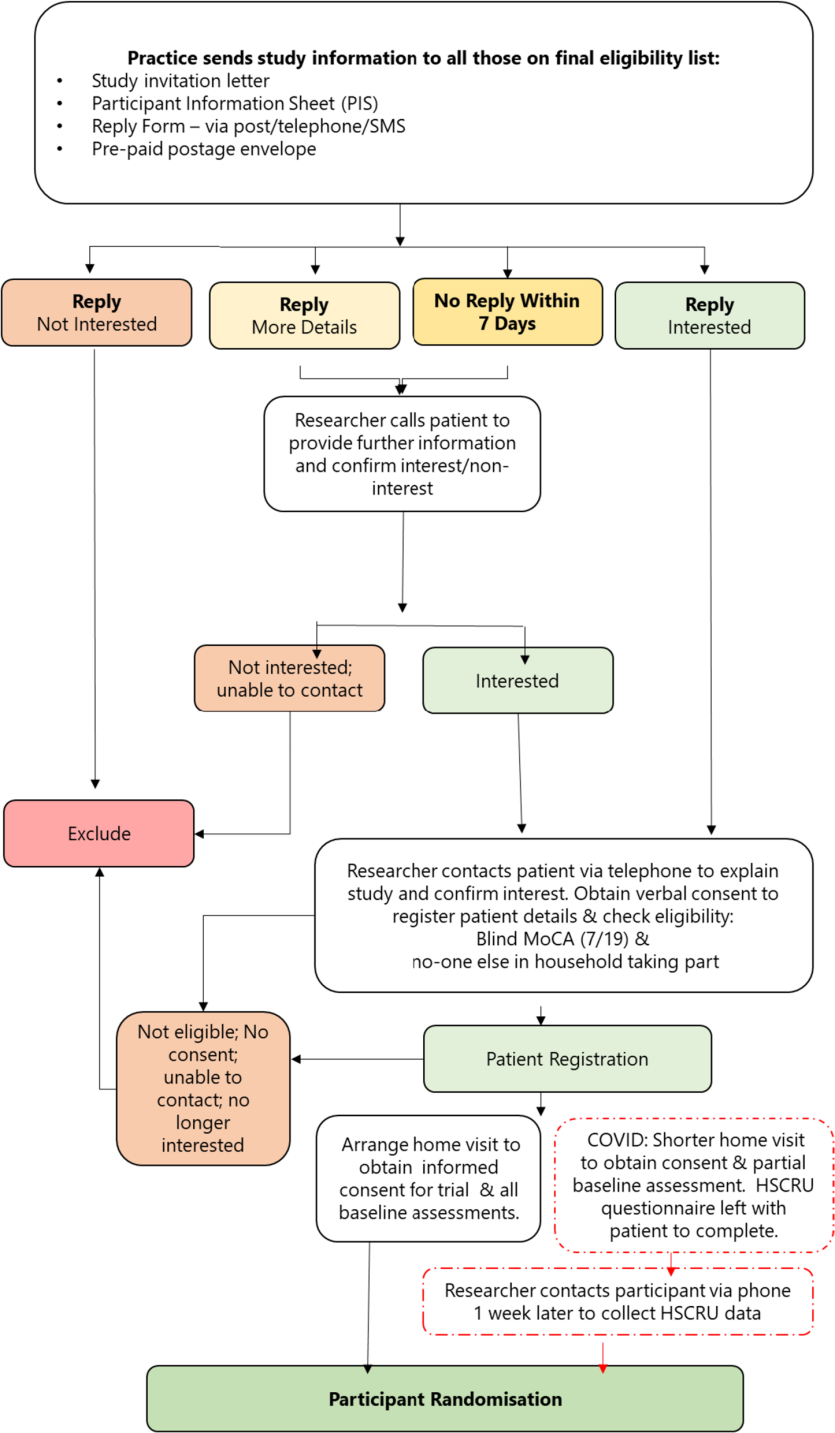
Participant recruitment flow diagram

Participants will be allocated to the treatment groups using the CTRU automated randomisation service. Individuals will be randomised with a 2.6:1 ratio (PROSPER intervention: usual care) using minimisation incorporating a random element, stratified by eFI, Nottingham Extended Activities of Daily Living (NEADL) score, GDS score, living arrangements, and practice deprivation index. All randomised participants will be informed of which trial arm they are allocated to via a letter from the CTRU. PICs will be notified of participants randomised to the intervention arm and will make contact to offer the PROSPER intervention. Researchers collecting baseline and follow-up data will be blinded to participant allocation. The process for recruitment and randomisation is outlined in Fig. 1.

### The PROSPER intervention

The PROSPER intervention was co-produced with our Intervention development Group, comprised six lay people and six primary care professionals. The PROSPER intervention will be delivered by a paid PIC worker. It is anticipated that the delivery teams will comprise 2.0 whole time equivalent (WTE) PIC workers, and 0.5 WTE team leader per geographical area. Wherever possible, delivery teams will work exclusively with an individual practice to maintain continuity.

#### Intervention delivery team training

Delivery team members will be trained in the intervention, which includes:

- An overview of frailty.
- General information on older people’s health conditions.
- Promoting the intervention in primary care.
- Opportunities to shadow primary care staff.
- Strengthening initial engagement with older adults.
- Identifying what is most important from the perspective of the older person for their health and wellbeing.
- Supporting change in older people using motivational interviewing and 11 specific behaviour change (BCT) techniques identified from an earlier literature review [17].
- Use of reflexive tools and ‘refresher’ sessions when delivery is underway.

#### Assessment of competency

Age UK teams will have a period of up to 4 weeks training, including organisational mandatory training, e.g. safeguarding, intervention, and trial procedure training. Key competencies such as Behavioural Change Techniques (BCT), ‘guided conversations’ and Motivational Interviewing (MI) will be assessed by the trainers. A video-recorded role play will also be used for reflective practice and competency assessment by members of the IDG before delivery teams ‘go live’. In addition, the PICs will be required to complete a reflective tool following their initial visits with two clients.

#### Intervention approach

Participants randomised to the intervention arm will receive a PROSPER service invitation letter and service summary information sheet from the PIC. This will be followed by a telephone call to discuss the service in more detail and arrange the first visit. Some particpants will also be asked to view a bespoke animation which outlines the intervention and its potential benefits. The efficacy of using the animation to enhance intervention uptake will be tested in a Study Within a Trial (SWAT). At the first visit, the PIC will facilitate a ‘guide conversation’ to ascertain the participant’s circumstance. Together the PIC and participant will identify any goals they wish to work towards and develop an action plan to achieve these. Underpinning this work will be SCT which highlights the importance of a sense of control and confidence to take on and persist with challenging tasks. Following the first visit, there will be two mandatory contacts: a 2-month review and the final ‘graduation’ visit after 12 weeks. The number of visits and types of activity undertaken during the 12-week period will depend on each individual participant. A full description of the intervention components can be seen in the TIDieR checklist [18].

#### Usual care (UC)

We define UC as ‘The wide range of care that is provided in a community whether it is adequate or not, without a normative judgement’ [19]. UC will be provided to both intervention and control participants using an unrestricted UC approach, whereby the trial protocol does not restrict access to UC, in line with our pragmatic trial design and the possible range of UC treatments available for older people with frailty [20]. UC is likely to be provided via GPs, community nurses and allied health professionals, and social service home care packages, but may also include use of voluntary sector services, day centres, and respite care. Use of services will be recorded at baseline and at 6- and 12-month follow-up assessments in both intervention and control groups and documented or collected via routine data. It is also anticipated that some participants will receive disease-specific care planning for single long-term conditions.

#### Contamination

Two potential sources of contamination will be monitored throughout the trial, although the risk is assumed to be low:

1. Healthcare professional (HCP) contamination. Practice staff have limited involvement with intervention delivery and will not receive detailed intervention training, reducing the risk of behaviour change approaches in UC participants. Access to the PCP PROSPER training manual will also be restricted to the delivery team and, during training, the importance of minimising contamination and mechanisms to limit contamination will be stressed.
2. Across allocation arm contamination. As the intervention is delivered in homes and communities, rather than at the practice or in clinics this is considered unlikely.

### Trial data collection

Data will be collected via paper Case Report Forms (CRFs) and questionnaires, or electronically via the CTRU registration/randomisation system, Remote Data Entry (RDE) database, and the electronic patient-reported outcome software ‘REDCap’. A full list of assessments and data collection tools is shown in Table 1. Assessments will be administered by research staff, or self-completed, supported by family or friends, if necessary. Research staff will receive training to ensure standardised completion as part of study initiation. The assessments will be ordered to prioritise primary outcome data. Participating general practices will be expected to maintain an electronic file of essential study documentation (eInvestigator Site File), which will be provided by CTRU. Any deviation in recording data outside of the protocol will be documented on the study records, together with the reason for their occurrence.

**Table 1:** Schedule of events

**PRIMARY AND HEALTH ECONOMICS DATA**							
			**Timeline**				
**Assessment**	**Type**	**Method of completion**	**Screening and initial eligibility check**	**Registration and final eligibility check**	**Baseline**	**6 months**	**12 months**
Participant screening	EHR data extract	Practice Staff	X				
Potential Participant Registration	Tel. consent /CRF	Research staff		X			
Consent/Consultee Declaration (Participant)	ICF	Self-completion (witnessed)			X		
Participant Demographics	CRF/ Online	Research staff			X		
Participant contact details	Online	Research staff			X		
COVID-19 Questions	CRF/Online	Research staff /Self-completion			X	X	X
Health Care Resource Use (inc. informal care, social care & out of pocket expenses)	Booklet/Online	Research staff / Self-completion			X	X	X
Montreal Cognitive Assessment (assessed at baseline visit or telephone call to register participant)	Booklet	Research staff		X	X		
SMAS-S (Self-management ability scale (short version))	Booklet/Online	Research staff / Self-completion			X	X^a^	X^a^
SF12 (short-form 12 items)	Booklet/Online	Research staff / Self-completion			X	X^a^	X^a^
EQ*5D-5L (EuroQol 5-Dimension 5 level)	Booklet/Online	Research staff / Self-completion			X	X^a^	X^a^
NEADL (Nottingham Extended Activities of Daily Life)	Booklet/Online	Research staff / Self-completion			X	X^a^	X^a^
GDS (5-item Geriatric Depression Scale)	Booklet/Online	Research staff / Self-completion			X	X^a^	X^a^
6-item De Jong-Gierveld loneliness scale	Booklet/Online	Research staff / Self-completion			X	X^a^	X^a^
Contamination	CRF	Age UK team/ Practice staff			X	X	X
Safety Reporting	CRF/ routine data	Age UK team/ CTRU			Ad-hoc	Ad-hoc	Ad-hoc
Primary and secondary Care Data (primary care contacts/A&E attendances/unplanned admissions/hospital bed stays/reason(s) for admission/outpatient attendances/care home admission/medications/mortality	CRF/ routine data	CTRU/ Practice Staff/ CRN			X		X
**INTERVENTION DATA**							
Assessment	Type	Method of completion	Training		Intervention period	Follow-up	
Training	CRF/ Obs.	PCP trainer	X				
Intervention delivered	RDE	Age UK team			X		
Competency Assessment (initial and ongoing)	Obs.	PE Researcher	X		X		
Sample of Age UK worker notes	Age UK notes	Age UK team member			At each contact		

^a^Self-competed at follow-up time point unless indicated that would prefer support to complete at baseline visit or do not return postal/electronic questionnaires

### Outcome measures

#### Baseline assessments

Baseline assessments *must* be completed before randomisation. The schedule of baseline assessments is shown in Table 1.

#### Follow-up assessments

Prior to follow-up, the CTRU will confirm the participants’ survival status and address via the NHS Digital Cohort service, if possible, or GP. Follow-up assessments will be completed at 6 and 12 months post-randomisation. Participants will be followed up by the CTRU via email for electronic completion or postal questionnaire. Telephone reminders (a maximum of 3 attempts over 1 month) will support this together with telephone completion/home visits if requested at baseline or if not returned within 2 weeks. We anticipate that approximately 15% of participants with physical disability and/or cognitive impairment may be less able to complete and return postal questionnaires [21]. All telephone and face-to-face follow-up will be by a researcher blind to allocation status where possible and independent of the practice and Age UK team. Participants will receive a ‘thank you’ and small unconditional monetary incentive (£10 gift voucher) at 12 months, distributed 2 weeks prior to the follow-up due date. All losses to follow-up, through death, withdrawal, and loss of contact will be fully reported. The schedule of follow-up assessments is shown in Table 1.

#### Use of routine data

Centrally available routine NHS data from NHS Digital (Hospital Episodes Statistics (HES)), Primary Care records (SystmOne, EMISWeb), and other data providers will be used to collect the following data to define UC and support economic evaluation:

- Primary care contacts
- A&E attendances
- Unplanned hospital admissions
- Hospital bed days
- Reason(s) for admission
- Outpatient attendances
- Care home admission
- Medications
- Mortality

To enable this, participants will be asked to consent to the research team sharing identifiable information with these data providers. Routine data will be sent to CTRU via a secure file transfer system.

### Sample size

One thousand three hundred thirty-seven participants (371 control arm, 966 intervention arm) provides 90% power at the 2.5% significance level (overall Type I error 5%) to detect an effect size of 0.30 in the primary outcomes of SF12 PCS and SF12 MCS scores (consistent with a minimum clinically important difference of 3 points and variance estimates based on published estimates for this population [22] and the unpublished results from our feasibility study). This accounts for 25% losses to follow-up and clustering by PICs in the intervention arm (15 PICs, 50 participants/PIC, 0.03 intraclass correlation coefficient (ICC), 0.30 variation in cluster size). Based on estimates of those 65+ years meeting eligibility criteria (20%), and those providing consent (20%), we anticipate that on average 60 participants will be recruited per practice.

### Statistical methods

#### Primary outcome analysis

PCP is designed to generate intervention effects on either mental or physical health-related quality of life, or both, depending on an individual’s personal circumstances. Social cognitive theory specifies factors that can profoundly affect both physical and mental health, so our decision to use the SF36 PCS and MCS as the co-primary outcome is closely aligned with our underpinning intervention theory [6] and logic model. Co-primary outcomes will therefore be the physical component summary (PCS) and mental component summary (MCS) scores of the SF12 as a measure of HRQoL at 12 months. Each primary outcome can, independently, characterise a clinically meaningful benefit of the intervention. The intervention will be considered effective if an improvement in either of the summary measures is demonstrated.

The primary analysis will compare mean PCS and MCS scores at 12 months between trial arms using partially nested mixed-effects linear regression models to account for clustering of outcomes in the intervention arm due to the PIC effects [23]. The models will be adjusted for the stratified design factors and participant level covariates expected a priori to be associated with the outcome of interest. The analysis will be undertaken on the intention to treat (ITT) population, which includes all randomised participants in their allocated treatment group, regardless of their level of treatment adherence. Estimated mean difference will be reported with 97.5% confidence intervals and p-values, together with unadjusted and adjusted ICC estimates. Model diagnostics will be used to check that the underlying assumptions are not violated in the analysis. We will explore missing data patterns and reasons for missingness to guide the assumptions around missing data. If it can be assumed data are missing at random (MAR) the primary Intention to Treat (ITT) analysis will use multiple imputation, enabling us to include all randomised participants in the primary ITT analysis. If the data cannot be assumed MAR, we will explore the use of other more complex methods for the primary analysis taking account of data missing not at random (MNAR), such as pattern mixture modelling.

#### Secondary outcome analysis

The binary secondary outcomes of care home admission, hospital admission, and mortality at 12 months will be compared between arms using logistic generalised estimating equations or random intercept models to account for heteroscedasticity [24] adjusted for stratification factors and participant level covariates associated with the outcome of interest. Continuous secondary outcomes of self-management ability scale score, NEADL score, 6-item De Jong Gierveld loneliness scale score and 5-item GDS score, obtained from participant questionnaire booklets at 12 months, will be analysed using the methods described for the primary outcome analysis.

Data on UC will be summarised descriptively. Exploratory mediator analyses will investigate whether intervention effects are mediated through key intervention mediators informed by the logic model (e.g. goals achieved by participant, self-management ability). Moderator analysis will explore whether the intervention effect depends on any baseline characteristics at the level of the cluster, i.e. PIC worker in the intervention arm, or participant. Moderation will be tested for via the inclusion of the proposed moderator variables (e.g. education level, level of frailty, living arrangements) alongside the interaction effect of treatment and moderator in the primary analysis model. We will also carry out sensitivity analyses using appropriate methods (such as CACE analysis) to assess the impact of the level of intervention delivered on the potential intervention effect. To assess the impact of death on our potential intervention effect, we will also repeat the primary analysis modelling but exclude those participants who have died. Finally, we will conduct a subgroup analysis to compare the effect of the intervention among those receiving and not receiving the SWAT intervention on the primary outcomes and the SWAT outcomes.

#### Data monitoring

Data will be monitored for quality and completeness by the CTRU. Missing data, from the PIC workers, will be chased until they are received, confirmed as not available, or when the study is at analysis. The CTRU/Sponsor will reserve the right to intermittently conduct source data verification exercises on a sample of participants, which will be carried out by staff from the CTRU/Sponsor. Source data verification will involve direct access to patient notes at the participating General Practice sites and the ongoing central collection of copies of consent forms.

### Health economics evaluation

The economic evaluation will compare the cost-effectiveness of PCP for older people with frailty with UC. In line with NICE guidance [25], the primary within-trial cost-effectiveness study will take the perspective of the health and personal social care sector. Analyses will report the differences in the cost of health and social care service utilisation between groups and the incremental cost-effectiveness ratios using quality-adjusted life years derived from the EQ-5D-5L.

A secondary analysis will take a societal perspective. Resource use will be collected through the trial CRFs and HES. The mean of these costs will be used as the unit cost estimate in the analysis. Sensitivity analyses will be undertaken to account for uncertainty. This will include (i) analysis using Quality Adjusted Life Years (QALYs) based on the SF6D, derived from the SF-12 and (ii) use of the non-parametric bootstrap method to produce a within-trial probabilistic sensitivity analysis of the incremental cost-effectiveness ratio (ICER). In addition to presenting the expected ICER and net monetary benefit (NMB), we will present the scatterplot on the cost-effectiveness plane, the 95% cost-effectiveness ellipse and the cost-effectiveness acceptability curve [26].

To assess the longer-term cost-effectiveness, we will use an analytical cost-effectiveness model, developed at an early stage of this research, and updated using data collected within the RCT. An updated literature search will be undertaken to ensure where data for the parameters is not available current evidence is utilised. Characterisation of uncertainty will rely on probabilistic evaluation either by bootstrapping directly from data available or by Monte Carlo simulation. Value of information analysis will be undertaken to characterise the burden of uncertainty on an NHS reimbursement decision maker or commissioner [27].

### Harms

In this patient population, acute illness resulting in hospitalisation, new medical problems, deterioration of existing medical problems and deaths are not unlikely. These adverse events (AE) and serious adverse events (SAE) will not be subject to expedited reporting to the main Research Ethics Committee (REC) but will be reported annually to the REC and reviewed at least bi-annually by relevant study oversight committees in accordance with the Trial Monitoring plan.

### Data management

#### Data storage and archiving post trial

All primary and secondary data including participant-reported questionnaires will be analysed and stored at the CTRU. The end of the trial is defined as 12 months post-randomisation of the last participant recruited to the trial. Both electronic and paper data will be securely archived at the University of Leeds for a minimum period of 10 years. Following authorisation from the Sponsor, arrangements for confidential destruction will then be made.

### Trial organisation and governance

#### Trial Steering Committee

The TSC will comprise an independent Chair, Health Economist, Statistician, and lay person. It will also include the PI, Programme Manager, and other co-applicants. A separate Data Monitoring and Ethics Committee (DMEC) is not required as the study is a psycho-social intervention with low risk of adverse events outside of those expected in a population of older adults. Instead, the TSC will fulfil this role, with the constitution of a sub-committee to review safety issues where this becomes necessary.

#### Programme Management Group (PMG)

The Programme Management Group (PMG) will oversee the whole programme of studies and is comprised the CI, Co-Applicants, and Co-investigators.

#### Trial Management Group (TMG)

The TMG, comprising the CI, CTRU team, other key external member of staff involved in the trial and a GP representative will be responsible for (i) protocol completion, (ii) CRF development, (iii) obtaining approval from the main REC and supporting applications for HRA Assessments, (iv) completing cost estimates and project initiation, (v) nominating members and facilitating the PSC, (vi) reporting of serious adverse events, (vii) monitoring of screening, recruitment, treatment and follow-up procedures and (viii) auditing consent procedures, data collection, trial end-point validation and database development.

#### Assessment and management of risk

Two main areas of risk have been identified:

1. Collection and linkage of potentially identifiable and confidential data

A key part of this study is successfully linking routine data extracted from primary care healthcare records with HES. A data management plan (DMP) will also define the data management procedures that will be followed to ensure patient data is secure.

(2) Vulnerable study population

This study targets older people with frailty and necessarily requires a sensitive approach to recruitment and consent. The research team has extensive experience of working with this potentially vulnerable group in community settings. In addition, on-going patient and public involvement (PPI) at every level [28] will ensure that study procedures are developed around the needs of older people with frailty. The study and procedures will comply with governance regulations, including ethical review, GCP, Mental Capacity Act 2005 and adhere to the UK policy framework for health and social care 2017.

## Discussion

The PROSPER trial will provide definitive evidence on the effects of PCP on quality of life for older people with frailty. Several mitigations were added to the protocol due to the COVID-19 pandemic, namely telephone screening and registration, use of the Blind MoCA, and remote REDCap data entry. These are intended to minimise burden on Practice staff, minimise burden and risks to potential participants, and reduce risk of disruption to trial processes in the event of further restrictions.

## Trial status

We are currently working to protocol V3.0 27/08/21. The first participant was recruited in May 2021. Data collection is on-going. The planned end date for recruitment is the 30/11/2024. Data analysis will commence after the follow-up period has been completed, and all data has been collected and verified.

### Supplementary Information

**Additional file 1.**

**Additional file 2.**

## Data Availability

Bradford Teaching Hospitals Foundation Trust (BTHFT) is the Data Controller. BTHFT will store non-trial data and the University of Leeds CTRU store trial data. Data will not be released prior to the end of the study, either for study publication or oral presentation purposes, without the permission of the TSC and subject to a data sharing agreement. During the archiving period, any requests for access to or copies of data will be considered by all collaborators in consultation. BTHFT will be the final arbiter of whether any disclosure/sharing should be agreed.

## Abbreviations

AE: Adverse event
CI: Confidence interval
CRN: Clinical Research Network
cRCT: Cluster randomised control trial
CRF: Case report form
CTRU: Clinical Trials Research Unit
eFI: Electronic Frailty Index
EQ5D: 5-level EQ-5D
GDS: Geriatric Depression Scale
GDPR: General Data Protection Regulations
GP: General Practitioner
HES: Health Episodes Statistics
ICC: Intraclass correlation coefficient
ICF: Individual consent form
ISRCTN: International Standard Randomised Controlled Trials Number
IRAS: Integrated Research Application System
ITT: Intention to treat analysis
LICTR: Leeds Institute of Clinical Trials Research
LTC: Long-term condition
LICTR: Leeds Institute of Clinical Trials Research
LIHS: Leeds Institute of Health Sciences
MCA: Mental Capacity Act
MCS: Mental Component Summary
MoCA: Montreal Cognitive Assessment
MDT: Multidisciplinary team
NEADL: Nottingham Extended Activities of Daily Living
NHS: National Health Service
NIHR: National Institute of Health Research
PCP: Personalised Care Plan(ning)
IPCP: Integrated Personalised Care Planning
PCS: Physical Component Summary
PHE: Public Health England
PIC: Personal Independence Coordinator
PMG: Programme Management Group
PSC: Programme Steering Committee
QoL: Quality of Life
QUALY: Quality of Life-Adjusted Year
RCT: Randomised controlled trial
RUSAE: Related unexpected serious adverse event
SAE: Serious adverse event
SCT: Social Cognitive Theory
SF-36: Short Form 36-item health questionnaire
TMG: Trial Management Group
TSC: Trial Steering Committee
UC: Usual care
UK: United Kingdom
