# Supplementary material for: PeRsOnaliSed care Planning for oldER people with frailty (PROSPER): protocol for a randomised controlled trial
Source: Trials. 2024 Jan 2;25:8. doi: 10.1186/s13063-023-07857-1 (PMC10759371; doi:10.1186/s13063-023-07857-1)
Supplement: Supplementary file 2 — Additional file 2. [file 13063_2023_7857_MOESM2_ESM.docx]

**:**

**The TIDieR (Template for Intervention Description and Replication) Checklist**

| **N^o^** | **What** | **Details** |
| --- | --- | --- |
| **1** | **Name** | PROSPER:  **PeRsOnaliSed care Planning for oldER people with frailty** |
| **2** | **Why: Rationale, theory, goal** | Frailty is a condition characterised by reduced biological reserves and increased vulnerability to adverse outcomes, including falls, disability, hospitalisation, and care home admission. Frailty has characteristics of considerable clinical importance, including higher reversibility at early stages than disability and higher predictive value than chronic disease for adverse outcomes.  **Personalised care planning**  Personalised Care Planning (PCP) is an anticipatory, negotiated series of discussions between a patient and a suitably trained individual to clarify goals, options and preferences and develop an agreed plan of action. The process aims to ensure that individual values and concerns shape how care is provided, instead of a focus on individual disease management processes. In PCP, shared decision making is a crucial mechanism, involving a collaborative discussion of treatment or management goals (goal setting) and developing an agreed plan for achieving these goals (action planning). Shared decision making enables linkage to additional mechanisms for improving outcomes through more effective self-management, better care coordination, and better access to community resources.  The full process includes creating the care plan and monitoring delivery of the plan through regular follow-up to help solve problems and achieve goals. Recognised key outcomes are improved physical and mental health, self-management capabilities, health-related behaviours and changes in health service use.  The process of PCP therefore enables effective, shared decision making, with identification of the personal outcomes of highest priority, and ongoing discussion to identify which strategies are most likely to achieve the identified outcomes. The process involves a move away from the reactive, crisis driven response currently evident in healthcare systems to a more proactive approach, considered highly relevant in frailty.  There are particular challenges to implementing and sustaining a complex intervention, such as PCP, in primary and community care settings. For example, there are well recognised pressures on general practice which constrain the capacity to take on new interventions and working practices. However, some of these pressures can also operate as levers for change. There is a growing recognition that siloed medical responses to multimorbidity may fail to address patient needs and preferences. There are further opportunities for alignment with emerging new models of care and efforts to reduce unplanned hospital admissions. It is therefore essential that promising interventions, such as PCP, are designed to be sufficiently robust and flexible to facilitate integration with commissioning and provider organisations.  PCP aims to ensure that people receive appropriate support for self-managing their condition. The theoretical background of PCP rests in principles of self-management developed in a number of theoretical models focusing on understanding factors influencing behaviour, including those that might help people make appropriate adaptations to improve health and ability to cope with illness and disability.  Social cognitive theory (SCT) is the theoretical model that resonates most with the tenets of PCP in the context of frailty, and provides the underpinning theory for developing and optimising our planned intervention. SCT specifies factors governing the acquisition of competencies that can profoundly affect physical and emotional wellbeing. It identifies knowledge, skills, self-efficacy, outcome expectations, goals and concrete plans, as well as the perceived social and environmental facilitators and impediments as core determinants influencing our health habits. Social and environmental factors are of core importance for maintaining quality of life in older age, so are highly relevant for optimising PCP for older people with frailty.  The focal determinant of SCT is self-efficacy because it is argued to affect health behaviour both directly and by its influence on the other determinants. SCT identifies the importance of a sense of control and confidence to take on and persist with challenging tasks. With this in mind the PCP intervention will target building confidence and providing older people with knowledge and skills to set personal goals, develop appropriate action plans and personalised problem-solving strategies. |
| **3** | **What Materials** | The key components of the intervention are the ‘Guided Conversation’ and ‘Graduation’ as delivered by the Personal Independence Co-ordinator (PIC).  A short (2 minute) standardised video will be shown to the older adult at the first visit. This will be used to illustrate the potential benefits of the PCP service and act as a ‘springboard’ for discussion.  A Summary Information Leaflet about the PCP service will be provided to the older adult reference purposes – this will contain key contact numbers.  Copies of action plans developed by the PIC and older adult will be left with the older adult if desired.  Additional information to support the older adult will be provided on a case by case basis as part of the behaviour change techniques used.  A package of blended training will be delivered close to delivery start dates and will focus on supporting delivery teams to effectively implement the intervention in practical terms. Along with specific Age UK organisational training (e.g. safeguarding and IG) it will include:   1. an overview of frailty 2. general information on older people’s health conditions 3. promoting the intervention in primary care 4. taking opportunities shadow primary care staff, e.g. community matron 5. strengthening initial engagement with older adults 6. identifying what is most important from the perspective of the older person for their health and wellbeing; 7. supporting change in older people through the use of motivational interviewing and behaviour change techniques 8. the use of reflexive tools and ‘refresher’ sessions when delivery is underway   Opportunities for accessing peer support from established teams delivering PCP will be discussed during training. This may include the use of a mobile App  The Intervention will be supported by a reference manual for use by the delivery team.  PICs will require smartcard enabled laptops for recording information in the Electronic Health care Records (EHR) and case management databases. The will also need to access web-based information about community assets. |
| **4** | **What Procedures** | Potential older adults (patients) will be targeted based on their frailty status as defined by the electronic Frailty Index (eFI). The target population will be aged 65 or over and have an eFI score of 0.21 or above. In addition, they will not be resident in a care home nor be registered on the Gold Standards Framework for end of life care.  First contact with suitable patients (potential older adults) will be made via an introductory letter branded with both the practice and Age UK logos, sent from the practice. The letter will be accompanied by an explanatory leaflet which summarises the service offer.  Unless the individual has contacted the surgery to ‘opt out’, the PIC should follow-up with a phone call 5-10 days after the initial letter has been sent out. The purpose of the phone call is to discuss the personalised care planning service in more detail and arrange a convenient time for the first visit.  Before the first visit the PIC will record a minimum amount of personal information from the patient’s EHR.  The first meeting should take place approximately one week after the initial phone call, if possible. The main focus of this visit will be on information sharing to make sure the older adult fully understands the intended purpose of the intervention and relationship building. This visit will;   - Make it clear that the service is time limited in order to manage expectations - Note any key contacts - Outline what support is and is not on offer and, importantly, when it is on offer i.e. Monday to Friday during office hours. This will include the use of a standardardised explanation and the video to illustrate potential benefits. - Leave the summary information booklet in for the older adult to read. An expected end date should be recorded in the booklet to remind the older adult that contact time is limited   A second visit will be schedule within one week. The focus of the second visit is to co-produce an action plan with older adult, through a ‘Guided Conversation’ which will incorporate ascertaining information about: a usual day; support networks; social networks; mobility and transport; health and fitness; safety and security; finances & paperwork and use of statutory support providers.  An action plan based on the older adults’ goals will co-produced with the older adult. The action plan will record: the goal; importance of the goal; confidence in achievement of the goal; motivation to achieve the goal; enablers to achieve the goal and the target date for achievement of the goal.  The PIC will work with the older adult to achieve the goals set out in the action plan.  Information about the older adult will be fed-back to the primary care team via a participants EHR (systmone, EMIS, VISION etc.). When necessary the information will be directed to a specific member of the primary care team. The PIC will be signposted to the appropriate member of clinical staff by a Practice Champion (a member of the primary care team with a special interest in older adults and/or frailty).  A review of the Action Plan will take place at two months. Older adults will be reminded of the expected end date of the intervention.  A final review will take place at 12 weeks. The current status of goal achievement will be recorded; delays in goal achievement due to third parties will be noted.  Older adults will ‘graduate’ from the service after a period of 12 weeks. If the intervention is deemed as no longer required by the older adult before the period has ended, PICs will employ ‘light touch’ contacts to review needs to allow the older adult time to reflect. The ‘graduation’ session provides an opportunity for the older adult and PIC worker to discuss future options including a step down in support and routes to re-engagement with services if necessary in the future. |
| **5** | **Who provided** | Screening for eligible older adults will be undertaken by administrative staff within general practices.  Potential older adults will be reviewed by GPs with in-depth knowledge of the practice list. All practice staff will have received proportionate GCP training to ensure adherence to the trial protocol.  Guided conversations and Graduations will be undertaken by Personal Independence Co-ordinators employed by local Age UK organisations (NB Age UKs are ‘brand partners’ i.e. they operate autonomously)  Support during the intervention period will be provided by the PIC. Support may also be provided by friends and family or other statutory or third sector organisations. |
| **6** | **How: mechanisms of delivery** | The intervention will be delivered by a trained team of Personal Independence Co-ordinators.  The intervention will be aimed at an individual but if appropriate an individual’s carer may be involved in the development of the action plan.  Specific behavioural change techniques will be employed to facilitate the older adult in achieving their goals. Specific BCTs identified as effective with this population are:   1. Social support (unspecified) 2. Goal setting (outcome) 3. Action planning 4. Information about health consequences 5. Problem solving 6. Credible source 7. Pharmacological support 8. Instruction on how to perform the behaviour 9. Verbal persuasion about capability 10. Review outcome goals 11. Biofeedback   Support will be offered face to face or via email and/or telephone, as appropriate.  Practices will allow PIC workers access to EHRs in order to record pertinent information for the primary care team.  Practices will identify key contacts to provide administrative and clinical support to the PIC. The latter will be expected to assist in providing opportunities for PICs to shadow members of the MDT. These staff will also be expected to encourage strong institutional commitment and ‘ownership’ within the primary care team by communicating the aims and objectives of PROSPER and how it might benefit patients. |
| **7** | **Where: location of delivery** | The intervention will be delivered in the participants own home, in communities and via the telephone and/or email, where appropriate |
| **8** | **When and how much** | The intervention will be delivered over a period of 12 weeks depending on the specific needs of the older adult.  Face to face contact will be made at the start (approximately 90 mins) and the end of the intervention (approximately 45 mins) with telephone follow up and hands on support in achieving goals given if necessary. A two month review of action plan progress will also be conducted face to face. |
| **9** | **Tailoring** | PCP is designed to be person centred and tailored to individual older adult needs and circumstances.  The Action Plan will remain a dynamic document and goals may be added/amended throughout the period of engagement. |
| **10*** | **Modifications** | The intervention has been modified in light of the process evaluation from the feasibility study. |
| **11** | **How well (planned)** | Fidelity will be determine by analysis of the intervention data (baseline to graduation):  Data will be captured on, for example, the number of contacts and sessions, date and duration of sessions and progression  An embedded process evaluation will explore any issues with implementation  Methods will include reviewing secondary data, non-participant observations, questionnaire surveys and semi-structure interviews |
| **12*** | **How well (actual)** | Ongoing |
